# ESGE Recommendations for Gynaecological Endoscopic Surgery for COVID-19 Outbreak

**Published:** 2020-05-07

## Background

The global coronavirus pandemic has become the dominant issue throughout the world whilst governments, nations and health services try to deal with its impact. Many countries are in either complete or partial lockdown to reduce the speed of transmission and save lives. Meanwhile healthcare systems are diverting their resources to looking after patients infected by the coronavirus. At the same time, women continue to present with gynaecological emergencies or are diagnosed with cancer, treatment of which cannot be postponed.

In order to guide our members and other colleagues to organise their priorities and minimise the risk to themselves and their patients, the ESGE Executive Board has decided to release this statement. It is anticipated that healthcare provision will gradually return to normal function, and that surgical priorities will change again to include elective procedures for benign conditions, as the pandemic gets under control.

- During the time of crisis healthcare providers need to be able to concentrate their resources on the care of people severely affected by the coronavirus; hence elective operations for benign conditions should not be carried out during the pandemic. Where possible, alternative medical treatment approaches should be considered to minimise suffering and keep women at home, away from hospitals.
- As the availability of PCR testing increases and healthcare systems start to control the coronavirus pandemic, it will be necessary to screen patients for coronavirus infection before planned surgical treatment.
- In suspected or documented COVID-19 positive patients, surgery should be postponed until full recovery, if there is no immediate life- threatening situation. Consideration should be given to non-surgical alternatives when possible. If this is not possible, surgery must be performed with full Personal Protective Equipment (PPE) (FFP3 or N95 standard respirator, visor, fluid repellent gown and gloves) worn by the entire theatre staff to reduce the risk of transmission.
- Hospitals should have arrangements in place to be able to look after women with gynaecological emergencies. Health services should also be able to care for women with possible gynaecological cancer and treat those who have been diagnosed with gynaecological cancer. Surgery for gynaecological cancer should continue, unless alternative interim options are possible until the end of the outbreak.
- Laparoscopic surgery for gynaecological emergencies and cancer is beneficial for the health system and society by reducing hospital stay and enabling quicker recovery, compared to open surgery. During laparoscopic surgery, the specific measures described below should be followed.
- Elective surgery should only be offered to disease free or recovered patients. The patient’s COVID-19 status should be evaluated using a combination of history (presence of symptoms), PCR testing (presence of infection), antibody testing (recovery after the disease) and chest x-ray examination, following national recommendations. For infected patients, surgery should be postponed until full recovery.

## Specific considerations for laparoscopic surgery

### 

There has been some concern regarding the safety of surgery related to the coronavirus infection. Current understanding is that surgery, both open or laparoscopic, is considered to carry a theoretical risk of transmission to healthcare professionals. Open surgery produces surgical smoke which may carry viral particles as discussed below and laparoscopic surgery is considered as an “aerosol” generating procedure (AGP) as well as producing surgical smoke. Risks of smoke inhalation to surgeons and staff when carrying out surgery, open or laparoscopic, have been documented. Laparoscopy might have some advantage over open surgery by confining the surgical smoke to a closed space, which gives the opportunity to control the release of smoke to the operating theatre more effectively and reduce exposure of the operating team.

Pneumoperitoneum is an essential component of laparoscopic surgery and brings the risk of aerosol exposure to the operating team. Aerosol exposure occurs during the intentional or unintentional release of CO_2_ which is used to achieve pneumoperitoneum during and at the end of laparoscopic surgery. CO_2_ release is more likely during insertion or removal of ports, introduction and removal of instruments through the ports, specimen retrieval and removal of pneumoperitoneum at the end of surgery. It is known that the COVID-19 virus is present in the blood of infected patients, but the viral load appears to be very low. In addition, it is known that surgical smoke contains viral particles such as HIV, HBV or HPV in infected patients. Currently, there is no data on the presence of COVID-19 in surgical smoke, but this is a possibility. Assuming that COVID-19 particles may be present within the body cavity of the patient being operated upon, there would be a risk to staff.

Therefore, based on very limited data and extrapolation from other viruses, additional risk of transmission from laparoscopic surgery is not clearly known but likely to be relatively low. COVID-19 is a respiratory virus and procedures which involve general anaesthesia are more likely to pose a much bigger transmission risk of the virus to the anaesthetic team.

Where laparoscopic surgery is carried out, the following precautions should be employed in addition to the general protection measures.

### Recommendations:

- All surgery should be considered high risk as asymptomatic patients may be carrying the virus. Preoperative testing should be considered.
- During laparoscopic surgery, take steps to minimise CO_2_ release.
- Close the taps of ports before inserting them to avoid escape of gas during insertion.
- Attach a CO_2_ filter that is capable of capturing viral particles to one of the ports for smoke evacuation if needed. Do not open the tap of any ports unless they are attached to a CO_2_ filter or being used to deliver the gas.
- Minimise introduction and removal of instruments through the ports as much as possible.
- For specimen retrieval such as in ectopic pregnancy, deflate the abdomen with a suction device before removing the specimen bag from the abdomen. Re-insert the port before turning CO_2_ on again.
- At the end of the procedure turn CO_2_ off, deflate the abdomen with a suction device and via the port with CO_2_ filter, before removal of the ports.
- Minimise use of ultrasound and diathermy if possible.
- Minimise sudden gas dispersal during total laparoscopic hysterectomy when the specimen is removed; deflate the abdomen with a suction device before removal the uterus through the vagina.

## Specific considerations for hysteroscopic surgery

### 

Hysteroscopy is not considered an AGP. In addition, limited evidence which is currently available does not indicate presence of the COVID-19 virus in genital fluids.

Use of electrosurgery during operative hysteroscopy does produce some surgical smoke (i.e. gaseous by-products of electrosurgery) but this mostly remains confined to the uterine cavity and is released through the outflow into the suction device or rapidly absorbed by the distention fluid media or into the circulation.

Hence, the significance of surgical smoke during hysteroscopic surgery is likely to be low or even absent. Hysteroscopic tissue removal systems have the advantage of lack of surgical smoke formation.

### Recommendations for hysteroscopic surgery:

- General protection measures before and during hysteroscopic surgery should be followed.
- Surgeons should wear standard droplet precaution PPE for hysteroscopic procedures; apron + gown, surgical mask, eye protection and gloves.
- Hysteroscopic surgery may be carried out as outpatient procedures without anaesthesia or as ambulatory procedures under anaesthesia. Regional anaesthesia or conscious sedation should be preferred over general anaesthesia to reduce the risk from intubation, if anaesthesia is required.
- Use of instruments that do not produce surgical smoke such as scissors, graspers and tissue retrieval systems should be preferred.
- Suction devices connected to the outflow sheaths of the operative hysteroscopes are highly recommended.

